# Transforming urban parks into comprehensive-health- promoting spaces

**DOI:** 10.3389/fpubh.2026.1784105

**Published:** 2026-02-18

**Authors:** Di Gan, Tao Wang

**Affiliations:** Health Engineering Management Research Institute, Tongji Hospital, School of Medicine, Tongji University, Shanghai, China

**Keywords:** city planning, green space, health promotion, public health, urban park

## 1 Introduction

The global urbanization rate reached 45% in 2025, more than double that of 1950 (1). As urbanization accelerates worldwide and population density in cities continues to increase, various health challenges have emerged. Noncommunicable diseases like heart disease, asthma, cancer and diabetes are made worse by unhealthy living and working conditions, inadequate green space, pollution such as noise, water and soil contamination, urban heat islands and a lack of space for walking, cycling and active living. Moreover, urbanization correlates with higher incidence of depression, anxiety, and mental illness (2). The quantity and quality of green space have been found to significantly correlate with cardiovascular disease, respiratory health, reproductive health, and even the probability of preterm birth (3–5). Meanwhile, multiple empirical studies have demonstrated the positive health effects of nature exposure: physical activity in blue spaces (natural water bodies) can enhance physical and mentalwellbeing; the mental wellbeing benefits of nature exposure can last up to 7 h; and childhood exposure to green space can reduce the risk of mental disorders by 55% (6–8). Therefore, expanding natural spaces such as green and blue spaces in cities represents a crucial solution to urban health challenges. Parks represent the perfect integration of nature and urban environments. The design and construction of urban parks oriented toward health promotion, rather than focusing solely on landscape aesthetics, should become a key research direction for urban planners and park designers.

## 2 Highlight health effects of park plants

The positive health effects of parks have been explained by some theories (9, 10). Research has further shown that even a 20-minute park visit can significantly enhance Subjective wellbeing (11). However, previous studies have focused only on the health impacts of green landscapes or the oxygen-enriching effects of plants, while overlooking the distinct roles of plant aromatic molecules in health promotion. According to Traditional Chinese Medicine herbology, almost all plants possess medicinal value: peppermint can refresh the mind and relieve headaches (12); ginkgo can regulate blood lipids and combat fatigue (13); honeysuckle has anti-inflammatory and antibacterial properties (14); and marigold can assist in treating respiratory diseases (15). These effects are gradually being confirmed by modern evidence-based medical research. Through multi-layered exposure to green space and medicinal plants, visitors can experience more efficient natural healing in urban parks. Therefore, health-promoting urban parks should incorporate plants' medicinal value and the city's primary health risks into species selection, combination, and replacement, building upon landscape design foundations.

## 3 Develop health monitoring functions of urban parks

Current research on the health effects of urban parks relies heavily on psychological scales, which are highly subjective and lack real-time capability, leaving room for improvement in scientific rigor. Advances in artificial intelligence, non-contact sensing devices, and digital twin technology now enable non-intrusive health monitoring of populations, making the smart upgrade of urban parks imperative (16). It is assumed that by deploying privacy-compliant, non-invasive, and imperceptible health monitoring devices in parks to remotely measure physiological data and detect facial micro-expressions, we can establish a more scientific and real-time way to observe and record the health status and the healing effects of parks. This facilitates real-time quantitative assessment of actual health benefits and comprehensive understanding of residents' physiological and psychological conditions, providing data support for precise allocation and scientific scheduling of public health resources. Meanwhile, the privacy of individuals needs to be thoroughly considered in monitoring parks. The ethical rules for public health monitoring should be addressed before implementation.

## 4 Expand social service functions in urban parks

Beyond static green spaces, landscapes, and trails, parks should incorporate more group activities and proactive social services. China's urbanization rate has surged from 43% to 67% in just two decades, posing major challenges to residents' health (17). The Chinese government prioritizes urban park development, launching national standard formulation for “Park City” construction in 2024 (18). Major metropolises like Beijing, Shanghai, Guangzhou, and Shenzhen have built “Cities of a Thousand Parks.” In China, parks have become cultural venues for group dancing, tai chi practice, and band performances during residents' leisure time. Some parks have also integrated health promotion and science education scenarios, disseminating Traditional Chinese Medicine health culture, transforming parks into multifunctional public spaces that integrate leisure, nature education, cultural experiences, and health promotion. This format provides residents with social and exercise atmospheres, enhances visitors' psychological sense of being valued and cared for, improves physical and mental health levels, and further increases their integration into urban life.

## 5 Strengthen multidisciplinary collaboration in urban park construction and development

The “Park+Great Health” concept is gradually becoming a critical research direction in urban development. Urban parks are evolving toward higher-level human-centered care and health support, representing a complex systematic project that requires integrating multidisciplinary knowledge—including medicine, landscape architecture, ecology, architecture, sociology, psychology, information technology, and management—to form collaborative synergies. Promoting partnerships between medical institutions and parks, where professional physicians participate in developing scientific standards and content review mechanisms for park-based health promotion services, while leveraging evidence-based medical research methods to scientifically quantify and systematically optimize health promotion effects (19). Strengthening the cultivation of interdisciplinary talent in landscape design and health to integrate health concepts throughout the entire park design process, from overall layout to detailed design. Jointly building health promotion park training bases with landscape design and management units to promote deep integration of theoretical exploration and practical innovation, forming a sustainable industry-education integration ecosystem for “Park+Great Health.”

## 6 Discussion

Natural environments have positive effects on human health. The construction of health-centered urban parks demonstrates significant development potential. Promoting the transformation of urban parks from landscape spaces to health-promoting integrated service venues represents an important pathway toward harmonious coexistence between humans and nature and the healthy co-development of residents and cities. Future interdisciplinary collaboration among landscape architecture, information technology, and medical professionals will provide more compelling theories, cases, and data for sustainable and healthy urban development.
